# Supplementary material for: Raman and X-ray Photoelectron Spectroscopic Study of Aqueous Thiol-Capped Ag-Zn-Sn-S Nanocrystals
Source: Materials (Basel). 2021 Jun 27;14(13):3593. doi: 10.3390/ma14133593 (PMC8269621; doi:10.3390/ma14133593)
Supplement: Supplementary file 1 [file materials-14-03593-s001.zip › materials-1258276-supplementary.pdf]

Supporting Information

Raman and X-ray Photoelectron Spectroscopic Study of Aqueous Thiol-Capped Ag-Zn-Sn-S Nanocrystals

Volodymyr Dzhagan[1,2], Oleksandr Selyshchev[3], Yevhenii Havryliuk[1,3], Nazar Mazur[1], Oleksandra Raievska[3,4,5], Oleksandr Stroyuk[6], Serhiy Kondratenko[2], A.P. Litvinchuk[7], M.Ya. Valakh[1], Dietrich R.T. Zahn[3,4*]

[1]V. Lashkaryov Institute of Semiconductors Physics, National Academy of Sciences of Ukraine, 03038 Kyiv, Ukraine
[2] *Physics Department, Taras Shevchenko National University of Kyiv, 60 Volodymyrs'ka str., 01601 Kyiv, Ukraine*
[3]Semiconductor Physics, Chemnitz University of Technology, D-09107 Chemnitz, Germany
[4]Center for Materials, Architectures, and Integration of Nanomembranes (MAIN), Chemnitz University of Technology, D-09107 Chemnitz, Germany
[5]L.V. Pysarzhevsky Institute of Physical Chemistry, Nat. Acad. of Science of Ukraine, 03028 Kyiv, Ukraine
[6]Forschungszentrum Jülich GmbH, Helmholtz-Institut Erlangen Nürnberg für Erneuerbare Energien (HI ERN), 91058 Erlangen, Germany
[7]Texas Center for Superconductivity and Department of Physics, University of Houston, Houston, TX 77204-5002, USA

*corresponding author, zahn@physik.tu-chemnitz.de

Details of the synthesis of AZTS NCs with varied composition.

$Ag_xCu_{1-x}ZnSnS_4$ (ACZTS) NC series

General protocol: 3 mL H_2O + 0.3 mL 0.5 M $SnCl_2$ in 4 M NaOH + 0.1 mL 5 M NH_4OH + 3.0 mL 1.0 M MAA + 0.15 mL 1.0 M $Zn(NO_3)_2$ + 3.0 mL (a mixture of different amounts of 0.1 M $AgNO_3$ + 0.1 M $Cu(NO_3)_2$) + 0.5 mL 1.0 M Na_2S. Heating: 5 min. *Note*: all solutions are prepared in DI water.

The following nominal compositions were prepared: $Ag_{30}Cu_0Zn_{15}Sn_{15}S_{50}$, $Ag_{25}Cu_5Zn_{15}Sn_{15}S_{50}$, $Ag_{20}Cu_{10}Zn_{15}Sn_{15}S_{50}$, $Ag_{15}Cu_{15}Zn_{15}Sn_{15}S_{50}$, $Ag_{10}Cu_{20}Zn_{15}Sn_{15}S_{50}$, $Ag_5Cu_{25}Zn_{15}Sn_{15}S_{50}$, $Ag_0Cu_{30}Zn_{15}Sn_{15}S_{50}$.

The as-prepared colloids were purified by adding 2.5 mL 2-propanol to 5.0 mL colloidal solution followed by the centrifugation (5 min at 5000 rpm). The precipitate was separated, dissolved in a minimal amount of DI water and then diluted with more DI water to 1.0 mL.

Variation of silver content - Ag_xZnSnS_4 NCs.

General protocol: (6-X) mL H_2O + 0.3 mL 0.5 M $SnCl_2$ in 4 M NaOH + 0.1 mL 5 M NH_4OH + 3.0 mL 1.0 M MAA + 0.15 mL 1.0 M $Zn(NO_3)_2$ + X mL 0.1 M $AgNO_3$ + 0.4 mL 1.0 M Na_2S. *Note*: all solutions are prepared in DI water. Heating: 7 min.

The following nominal compositions were prepared:

$Ag_0Zn_{15}Sn_{15}S_{40}$ (X = 0)
$Ag_5Zn_{15}Sn_{15}S_{40}$ (X = 0.5 mL)
$Ag_{10}Zn_{15}Sn_{15}S_{40}$ (X = 1.0 mL)
$Ag_{15}Zn_{15}Sn_{15}S_{40}$ (X = 1.5 mL)
$Ag_{20}Zn_{15}Sn_{15}S_{40}$ (X = 2.0 mL)
$Ag_{25}Zn_{15}Sn_{15}S_{40}$ (X = 2.5 mL)
$Ag_{30}Zn_{15}Sn_{15}S_{40}$ (X = 3.0 mL)

The as-prepared colloids were purified by adding 5 mL 2-propanol to 10 mL colloidal solution followed by the centrifugation (5 min at 5000 rpm). The precipitate was separated, dissolved in a minimal amount of DI water and then diluted with more DI water to 1.0 mL.

Variation of zinc content - $Ag_2Zn_xSnS_4$ NC.

General protocol: 3 mL H_2O + 0.3 mL 0.5 M $SnCl_2$ in 4 M NaOH + 0.1 mL 5 M NH_4OH + 3.0 mL 1.0 M MAA + X mL 1.0 M $Zn(NO_3)_2$ + 3.0 mL 0.1 M $AgNO_3$ + 0.4 mL 1.0 M Na_2S. *Note*: all solutions are prepared in DI water. Heating: 10 min.

The following nominal compositions were prepared:

$Ag_{30}Zn_0Sn_{15}S_{40}$ (X = 0)
$Ag_{30}Zn_5Sn_{15}S_{40}$ (X = 0.5 mL)
$Ag_{30}Zn_{10}Sn_{15}S_{40}$ (X = 1.0 mL)
$Ag_{30}Zn_{15}Sn_{15}S_{40}$ (X = 1.5 mL)
$Ag_{30}Zn_{20}Sn_{15}S_{40}$ (X = 2.0 mL)
$Ag_{30}Zn_{25}Sn_{15}S_{40}$ (X = 2.5 mL)
$Ag_{30}Zn_{30}Sn_{15}S_{40}$ (X = 3.0 mL)

The as-prepared colloids were purified by adding 5 mL 2-propanol to 10 mL colloidal solution followed by the centrifugation (5 min at 5000 rpm). The precipitate was separated, dissolved in a minimal amount of DI water and then diluted with more DI water to 1.0 mL.

Variation of tin content - $Ag_2ZnSn_xS_4$ NC.

General protocol: 3 mL H_2O + X mL 0.5 M $SnCl_2$ in 4 M NaOH + 0.1 mL 5 M NH_4OH + 3.0 mL 1.0 M MAA + 0.15 mL 1.0 M $Zn(NO_3)_2$ + 3.0 mL 0.1 M $AgNO_3$ + 0.4 mL 1.0 M Na_2S. *Note*: all solutions are prepared in DI water. Heating: 7 min.

The following nominal compositions were prepared:

$Ag_{30}Zn_{15}Sn_0S_{40}$ (X = 0.25 mL)
$Ag_{30}Zn_{15}Sn_{2.5}S_{40}$ (X = 0.25 mL)
$Ag_{30}Zn_{15}Sn_{5.0}S_{40}$ (X = 0.35 mL)
$Ag_{30}Zn_{15}Sn_{7.5}S_{40}$ (X = 0.40 mL)
$Ag_{30}Zn_{15}Sn_{10}S_{40}$ (X = 0.45 mL)
$Ag_{30}Zn_{15}Sn_{12.5}S_{40}$ (X = 0.50 mL)
$Ag_{30}Zn_{15}Sn_{15}S_{40}$ (X = 1.00 mL)

The as-prepared colloids were purified by adding 2.5 mL 2-propanol to 5.0 mL colloidal solution followed by the centrifugation (5 min at 5000 rpm). The precipitate was separated, dissolved in a minimal amount of DI water and then diluted with more DI water to 1.0 mL.

Variation of sulfur content - Ag_2ZnSnS_x NCs.

General protocol: 3 mL H_2O + 0.3 mL 0.5 M $SnCl_2$ in 4 M NaOH + 0.1 mL 5 M NH_4OH + 3.0 mL 1.0 M MAA + 0.15 mL 1.0 M $Zn(NO_3)_2$ + 3.0 mL 0.1 M $AgNO_3$ + X mL 1.0 M Na_2S. *Note*: all solutions are prepared in DI water. Heating: 5 min.

The following nominal compositions were prepared:

$Ag_{30}Zn_{15}Sn_{15}S_{25}$ (X = 0.25 mL)
$Ag_{30}Zn_{15}Sn_{15}S_{30}$ (X = 0.25 mL)
$Ag_{30}Zn_{15}Sn_{15}S_{35}$ (X = 0.35 mL)
$Ag_{30}Zn_{15}Sn_{15}S_{40}$ (X = 0.40 mL)
$Ag_{30}Zn_{15}Sn_{15}S_{45}$ (X = 0.45 mL)
$Ag_{30}Zn_{15}Sn_{15}S_{50}$ (X = 0.50 mL)
$Ag_{30}Zn_{15}Sn_{15}S_{100}$ (X = 1.00 mL)

The as-prepared colloids were purified by adding 2.5 mL 2-propanol to 5.0 mL colloidal solution followed by the centrifugation (5 min at 5000 rpm). The precipitate was separated, dissolved in a minimal amount of DI water and then diluted with more DI water to 1.0 mL.

Variation of both silver and zin content - $Ag_xZn_ySnS_4$ NCs.

General protocol: 3 mL H_2O + 0.3 mL 0.5 M $SnSO_4$ in 4 M NaOH + 0.1 mL 5 M NH_4OH + 3.0 mL 1.0 M MAA + X mL 1.0 M $Zn(NO_3)_2$ + 2X mL 0.1 M $AgNO_3$ + 0.4 mL 1.0 M Na_2S. *Note*: all solutions are prepared in DI water. Heating: 10 min.

The following nominal compositions were prepared:

$Ag_0Zn_0Sn_{15}S_{40}$ (X = 0)
$Ag_5Zn_{2.5}Sn_{15}S_{40}$ (X = 0.025 mL)
$Ag_{10}Zn_5Sn_{15}S_{40}$ (X = 0.050 mL)
$Ag_{15}Zn_{7.5}Sn_{15}S_{40}$ (X = 0.075 mL)
$Ag_{20}Zn_{10}Sn_{15}S_{40}$ (X = 0.100 mL)
$Ag_{25}Zn_{12.5}Sn_{15}S_{40}$ (X = 0.125 mL)
$Ag_{30}Zn_{15}Sn_{15}S_{40}$ (X = 0.150 mL)

The as-prepared colloids were purified by adding 2.5 mL 2-propanol to 5.0 mL colloidal solution followed by the centrifugation (5 min at 5000 rpm). The precipitate was separated, dissolved in a minimal amount of DI water and then diluted with more DI water to 1.0 mL.

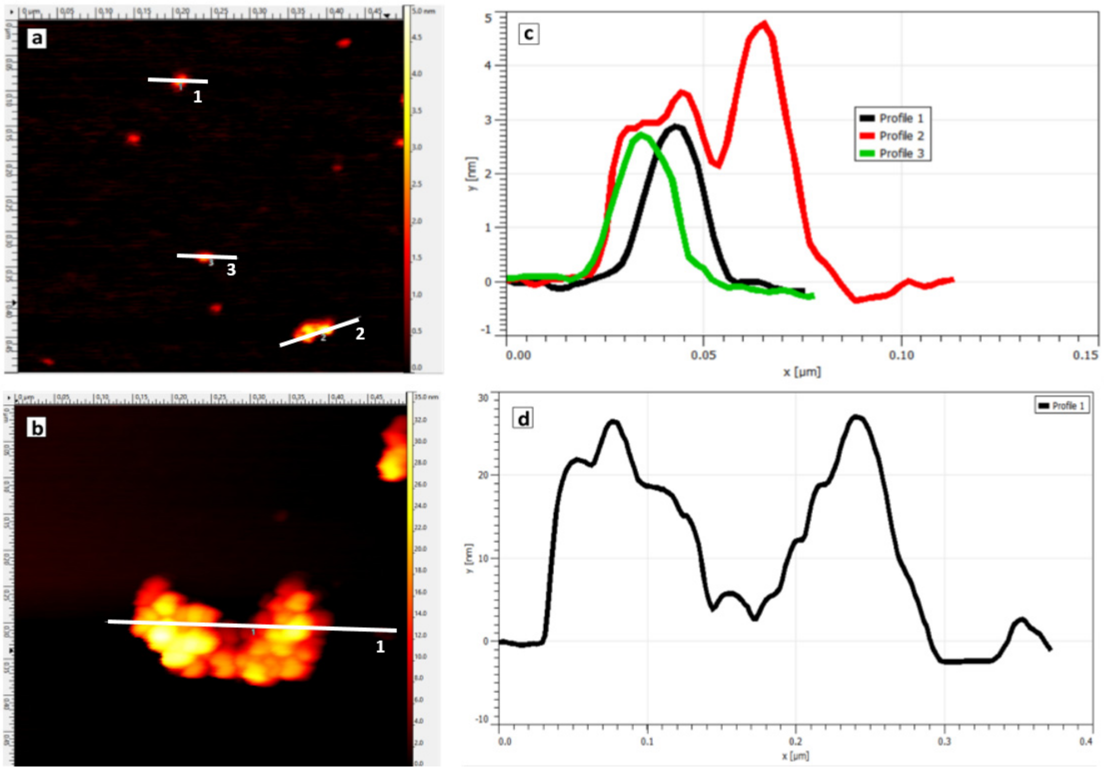

Figure S1. AFM images (a,b) and profiles (c,d) of separate AZTS NCs (a,c) and a NC aggregate (b,d).

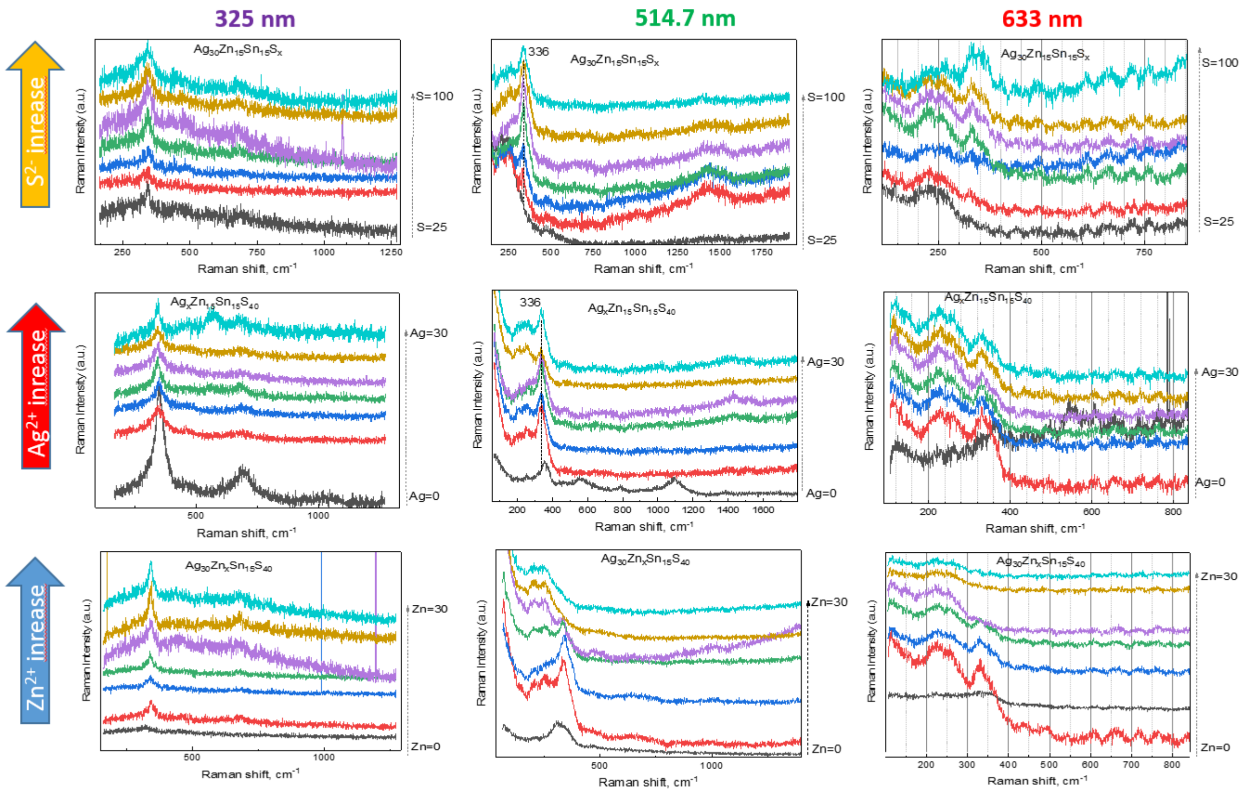

Figure S2. A comparative plot of normalized Raman spectra of S-, Ag, and Zn-series of Ag-Zn-Sn-S NCs at λ_{exc}=325 nm, 514.7 nm (b), and 633 nm (c).

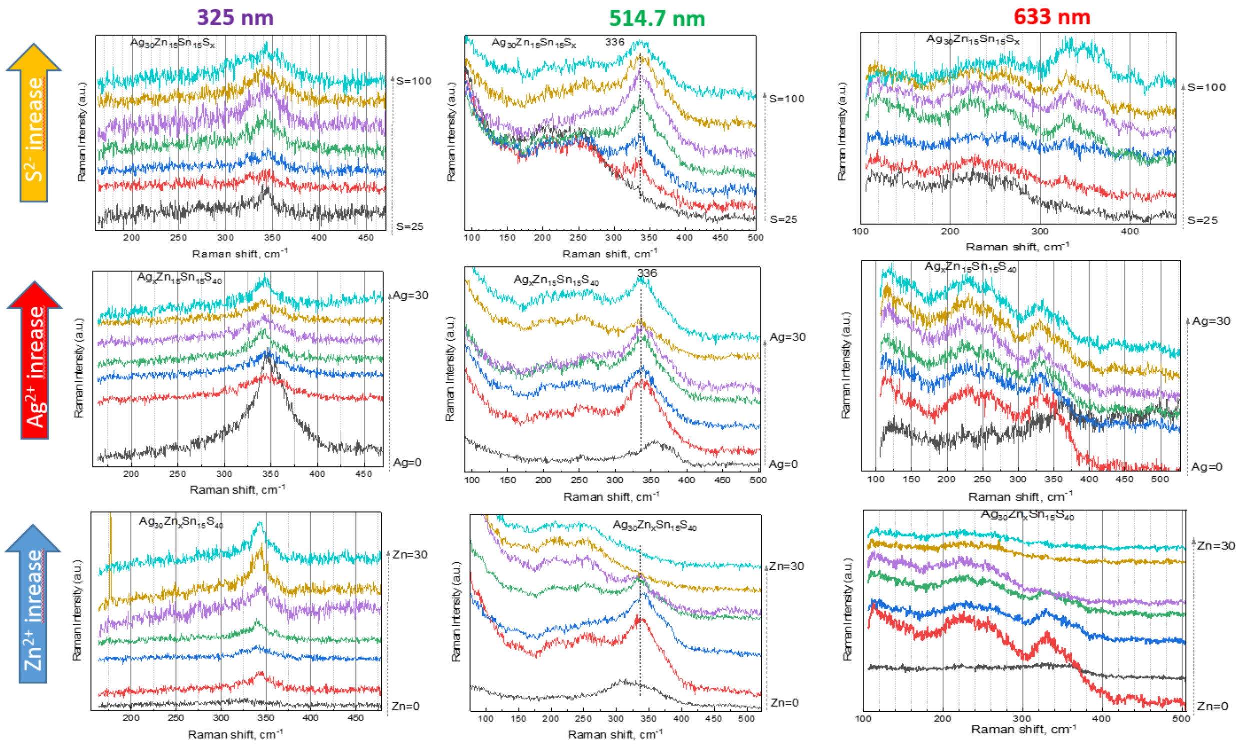

Figure S3. A comparative plot of as-measured Raman spectra of S-, Ag, and Zn-series of Ag-Zn-Sn-S NCs at λ_{exc}=325 nm, 514.7 nm (b), and 633 nm (c).

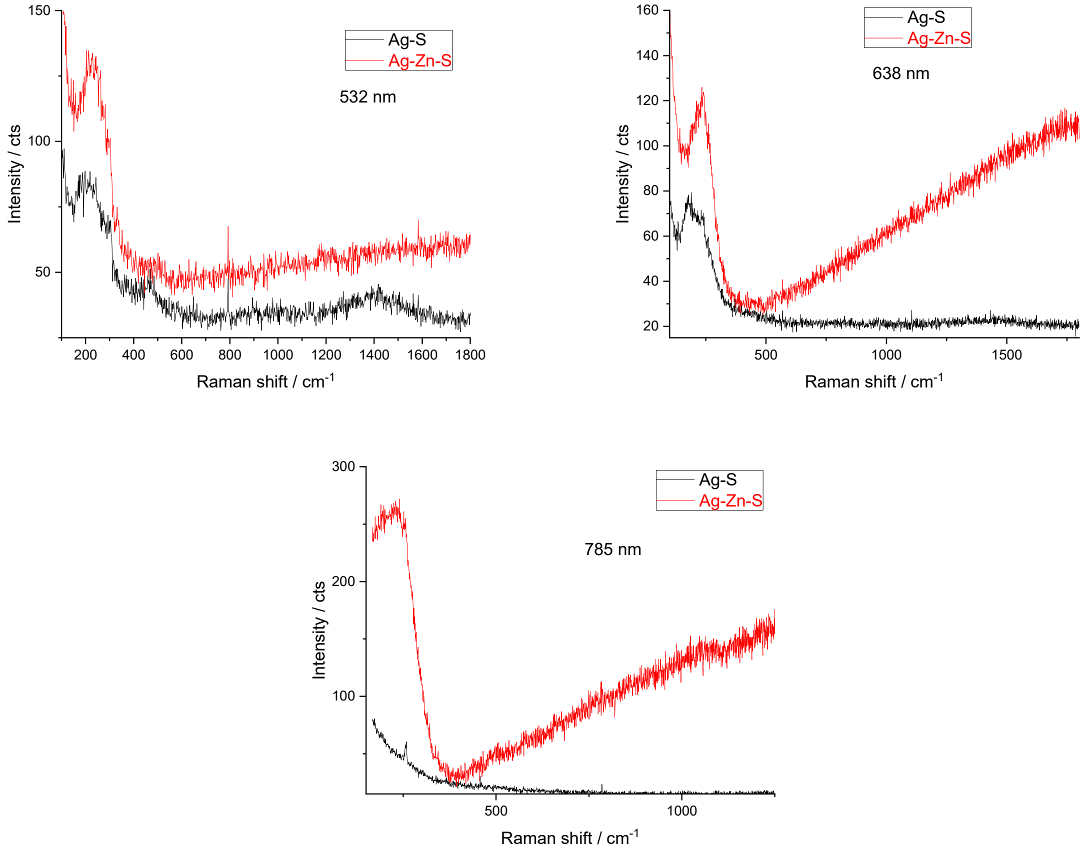

Figure S4. Raman spectra of Ag-S and Ag-Zn-S NCs at λ_{exc}=532 nm (a), 638 nm (b), and 785 nm (c).

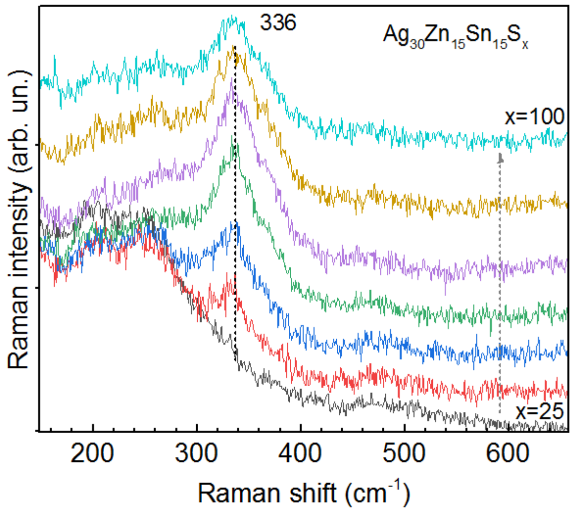

Figure S5. Raman spectra of Ag_2ZnSnS_x NC series (λ_{exc}=514.7 nm).

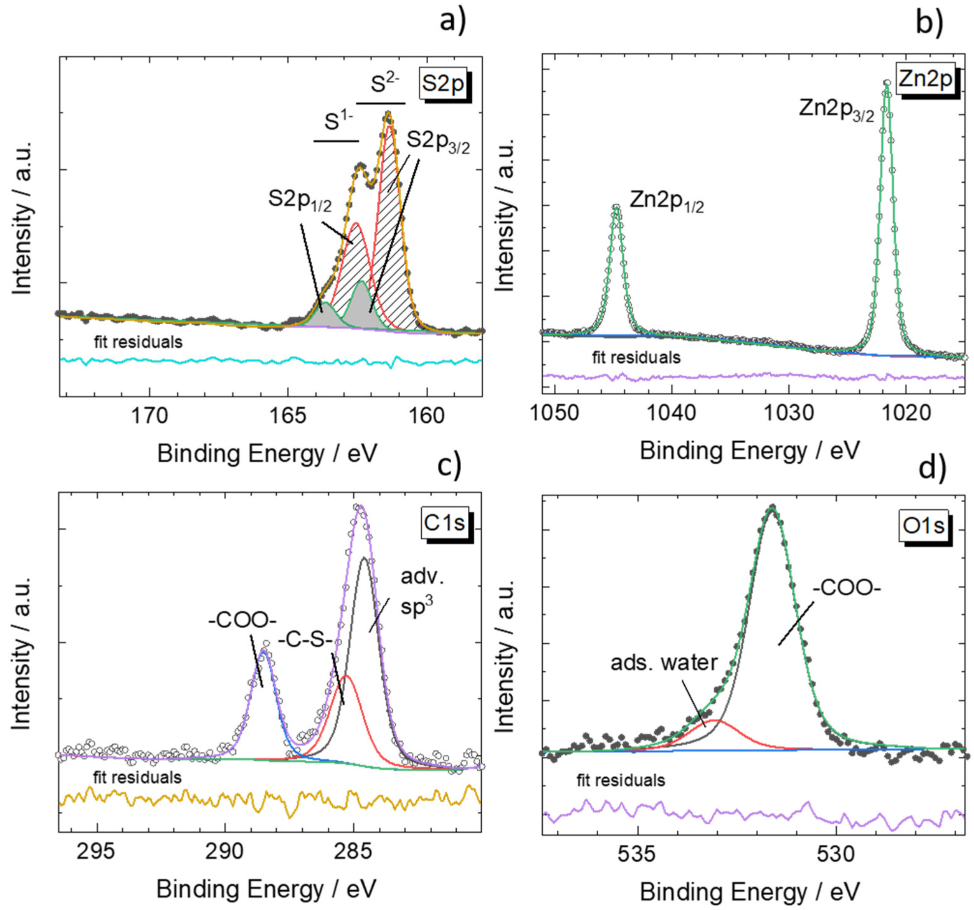

Figure S6. Representative high-resolution XPS spectra of Ag_2ZnSnS_x NCs in the range of S2p (a), Zn2p (b), C1s (c), O1s (d) orbitals.
